# Microbial Interventions for Inflammatory Skin Diseases: A Systematic Review and Meta-Analysis of Atopic Dermatitis and Psoriasis

**DOI:** 10.3390/microorganisms13112416

**Published:** 2025-10-22

**Authors:** Yamil Liscano, Daniel Muñoz Morales, Fernanda Suarez Daza, Sinthia Vidal Cañas, Darly Martinez Guevara, Esteban Artunduaga Cañas

**Affiliations:** 1 Grupo de Investigación en Salud Integral (GISI), Department of Health Sciences Faculty, Universidad Santiago de Cali, Cali 760035, Colombia; dmmdaniel456@gmail.com (D.M.M.); fernandasuarezdaza@gmail.com (F.S.D.); sinthia.vidal00@usc.edu.co (S.V.C.); darly.martinez00@usc.edu.co (D.M.G.); 2 Programa de Medicina, Universidad Central del Valle del Cauca (UCEVA), Tuluá 763533, Colombia; estebanartunduaga@live.com

**Keywords:** dermatology, probiotics, prebiotics, synbiotics, postbiotics, microbiome, meta-analysis, dysbiosis

## Abstract

Inflammatory dermatological diseases represent a significant global health burden, with emerging evidence suggesting that modulation of the gut–skin axis microbial interventions may offer therapeutic benefits. However, current evidence is fragmented, with considerable heterogeneity limiting definitive conclusions. A systematic review and a meta-analysis were conducted following PRISMA guidelines, registered in PROSPERO (CRD42024629809). Seven databases were searched for randomized controlled trials evaluating probiotics, synbiotics, or postbiotics in inflammatory skin conditions. Primary outcomes included disease severity scores (SCORAD for atopic dermatitis, PASI for psoriasis). Statistical analysis employed random-effect models with standardized mean differences (SMDs) and Hedges’ g as effect size measures, using R software. Heterogeneity among studies was assessed using Q statistics and the I^2^ index. Results: In total, 19 studies encompassing 1104 participants met the inclusion criteria. For atopic dermatitis, a meta-analysis of 12 studies (n = 817) demonstrated significant clinical improvement with microbial interventions versus placebo (SMD = −0.72; 95% CI: −1.26 to −0.17; *p* = 0.015), though substantial heterogeneity in the treatment effects was observed across studies (I^2^ = 85.1%). The psoriasis results were more variable, with five studies (n = 287) showing non-significant pooled effects (SMD = −0.63; 95% CI: −1.74 to 0.48; *p* = 0.192). Multi-strain formulations and synbiotic combinations appeared to show greater efficacy compared to single-strain preparations. Safety profiles remained consistently favorable across all interventions. Microbial interventions represent a promising adjunctive therapeutic approach for inflammatory dermatological diseases, particularly atopic dermatitis, acting via gut–skin axis mechanisms. The substantial heterogeneity between the included studies emphasizes the need for standardized protocols and personalized medicine approaches integrating microbiome profiling to optimize clinical outcomes.

## 1. Introduction

Dermatological diseases constitute a diverse group of conditions affecting the skin, its appendages, and subcutaneous tissue. This spectrum includes pathologies of infectious, inflammatory–autoimmune, neoplastic, and allergic origin, as well as chronic and congenital conditions influenced by environmental factors [1,2]. Furthermore, the skin is a key organ for the manifestation of underlying systemic diseases, such as HIV infection and various metabolic disorders [3,4,5].

Chronic inflammatory conditions like atopic dermatitis, psoriasis and acne vulgaris represent a significant burden on healthcare systems worldwide and cause substantial morbidity, with a profound impact on individuals’ quality of life [6,7]. Although they do not align with the classic autoimmune disease, one of their main characteristics is chronic inflammation. In this context, the study of the skin microbiome has gained exceptional relevance due to its close relationship with the maintenance of immunological homeostasis, the integrity of the epidermal barrier, and the modulation of inflammatory processes [8,9,10]. This understanding has spurred interest in therapeutic interventions aimed at modulating the microbiota. Among such therapeutic interventions, a range of microbial-based strategies, including probiotics (live microorganisms), synbiotics (synergistic combinations), postbiotics (inactivated microorganisms, peptides and their metabolites), and even topical live biotherapeutics, have been positioned as promising strategies for the prevention and treatment of skin diseases [11,12,13]. Notably, postbiotic peptides derived from microbial metabolism can function as antimicrobial agents, wound-healing promoters, and immunomodulatory compounds (offering additional therapeutic mechanisms beyond live microbial interventions), and may provide enhanced stability, targeted delivery, and reduced risk of adverse effects compared to live probiotic formulations while maintaining beneficial effects on skin barrier function and inflammatory responses [14,15,16,17].

Preliminary studies have documented symptomatic improvements in patients with atopic dermatitis, psoriasis, and acne treated with these formulations [8,18]. However, the current clinical evidence on the efficacy of these therapies is inconclusive and marked by significant heterogeneity. There is considerable variability in the strains used, administration routes (oral vs. topical), dosages, study populations, and outcomes measured. This diversity has led to conflicting results, making it difficult to draw firm conclusions about their overall efficacy and complicating their translation into clinical practice [11]. This lack of consensus underscores the need for a rigorous synthesis of the available evidence.

Therefore, the objective of this systematic review and meta-analysis is to synthesize and critically evaluate the available evidence on the efficacy and safety of microbial-based interventions (including probiotics, synbiotics, and postbiotics) in the treatment of inflammatory dermatological conditions, with a focus on atopic dermatitis and psoriasis. Through this analysis, we aim to clarify their therapeutic potential by quantitatively assessing their impact on disease severity, identify the most effective interventions, and guide the design of future research in the field of microbiome-based therapies. While previous reviews exist, this meta-analysis provides an updated synthesis focused specifically on atopic dermatitis and psoriasis, including recently published trials and a comprehensive assessment of probiotics, synbiotics, and postbiotics, aiming to clarify the conflicting evidence in these two distinct conditions.

## 2. Materials and Methods

### 2.1. Study Protocol

This review was carried out using the Cochrane Collaboration Handbook tools [19] with the PRISMA [20] guides, and it was first registered in PROSPERO (accessed on 19 December 2024) under the code CRD42024629809. Based on the above, the PICO question was designed.

### 2.2. Research Question

The primary research question was structured using the PICO framework: In patients with inflammatory dermatological conditions (P), are microbial-based interventions such as probiotics, prebiotics, or synbiotics (I), compared to placebo or standard care (C), effective in improving clinical and patient-reported outcomes (O)? Key outcomes of interest included disease severity, skin barrier function, quality of life, and safety.

### 2.3. Eligibility Criteria

#### 2.3.1. Inclusion Criteria

Studies were deemed eligible for inclusion if they met the following specifications: (1) Eligibility was restricted to randomized controlled trials (RCTs). (2) No limitations were placed on the specific experimental design (e.g., parallel, crossover) or the duration of the follow-up period. (3) The review included studies of human participants of any age diagnosed with inflammatory dermatological conditions. (4) The intervention involved evaluating the effectiveness of prebiotics, probiotics, synbiotics, or topical microbiome-based therapies. (5) Studies with multiple treatment arms were included, provided that the data corresponding to the microbiome-based intervention could be distinctly isolated and analyzed. (6) Publications were limited to those available in Portuguese, Spanish, and English. (7) No restrictions were applied to the strain(s) used (single or combined), the formulation (e.g., capsule, topical cream, gel), administration duration, or treatment regimen. (8) To be included, an article was required to report on at least one of the following outcome domains:

- Clinical efficacy: investigator-assessed improvement, disease severity scores (e.g., SCORAD, EASI, PASI, IGA), time to clinical response, or prevention of recurrence.
- Physiological markers: indicators of skin barrier function (e.g., TEWL, stratum corneum hydration) or inflammatory biomarkers.
- Patient-reported outcomes: quality of life measures (e.g., DLQI, CDLQI).
- Microbiological parameters: data on microbiome diversity.
- Safety: the incidence of adverse events.

#### 2.3.2. Exclusion Criteria

Studies were excluded based on any of the following conditions: (1) Articles published as preprints, conference abstracts, or letters to the editor with insufficient primary data were not considered. (2) Studies for which the full-text article could not be retrieved were excluded. (3) Publications reporting on duplicate or overlapping patient cohorts from a single primary investigation were excluded. (4) Non-human studies, including those based on animal models, were ineligible. (5) Studies involving participants with congenital or acquired immunodeficiencies, concurrent systemic autoimmune diseases, or chronic systemic conditions that could interfere with skin microbiome assessment were excluded. (6) Studies involving co-interventions where the specific contribution of the microbiome-based therapy could not be distinguished from the effects of other simultaneous dermatological interventions were excluded. Additionally, trials that failed to specify the microbial strain(s) present in the supplied product or that focused exclusively on cosmetic outcomes without assessing a clinical dermatological endpoint were also ineligible.

### 2.4. Data Sources and Search Strategy

The search was conducted in the following databases: PubMed, EMBASE, LILACS, SCOPUS, Web of Science, Science Direct, and Cochrane Clinical Trials. Filters for Spanish, Portuguese, and English were applied, with date filters applied where necessary. The search strategy was designed and implemented from January 2025 to March 2025 by three independent researchers (Y.L.*, S.V.C., E.A.C.), using the following strategy:

(“probiotics” OR “gut microbiota” OR “skin microbiome” OR “cutaneous microbiota” OR “microbiome” OR “synbiotics” OR “prebiotics” OR “postbiotics” OR “topical probiotics” OR “microbiome-based therapy”) AND (“atopic dermatitis” OR “eczema” OR “acne vulgaris” OR “acne” OR “psoriasis” OR “seborrheic dermatitis” OR “rosacea” OR “skin barrier function” OR “dermatological condition” OR “inflammatory skin disease” OR “skin inflammation”) AND (“treatment” OR “therapy” OR “clinical outcome” OR “efficacy” OR “therapeutic effect” OR “management” OR “intervention” OR “SCORAD” OR “EASI” OR “PASI” OR “IGA” OR “DLQI”) AND (“randomized controlled trial” OR “clinical trial” OR “RCT” OR “controlled trial” OR “randomized trial”).

To ensure a comprehensive literature search, our primary database queries, which were adapted for each database’s unique requirements, were supplemented by reviewing the bibliographic references of pertinent articles and conducting a manual web search. If a study lacked complete information, we sought supplementary data from its clinical trial registration, consulting either ClinicalTrials.gov (https://clinicaltrials.gov/, accessed between 15 January and 20 March 2025) or the repository designated in the article. For data storage and handling, we employed Rayyan—Intelligent Systematic Review (https://www.rayyan.ai/, accessed between 15 January and 30 March 2025).

### 2.5. Selection and Data Extraction

Two reviewers (D.M.G., F.S.D.) independently screened titles, abstracts, and then full texts for eligibility. Corresponding authors were contacted for inaccessible articles. Selection discrepancies were resolved by consensus, with a third reviewer (Y.L.) who adjudicated any remaining disagreements. Inter-rater agreement was quantified using the Cohen’s Kappa statistic.

Data extraction was performed independently by three reviewers (S.V.C., E.C., D.M.M.) using a standardized form covering: study characteristics, participant demographics, intervention details, and outcome measures. Two additional reviewers (F.S.D., D.M.G.) subsequently verified the accuracy and integrity of all extracted information. Inter-rater agreement for each stage of the selection process was quantified using Cohen’s Kappa statistic, with the final coefficients reported alongside the PRISMA flow diagram.

**Figure 1:**
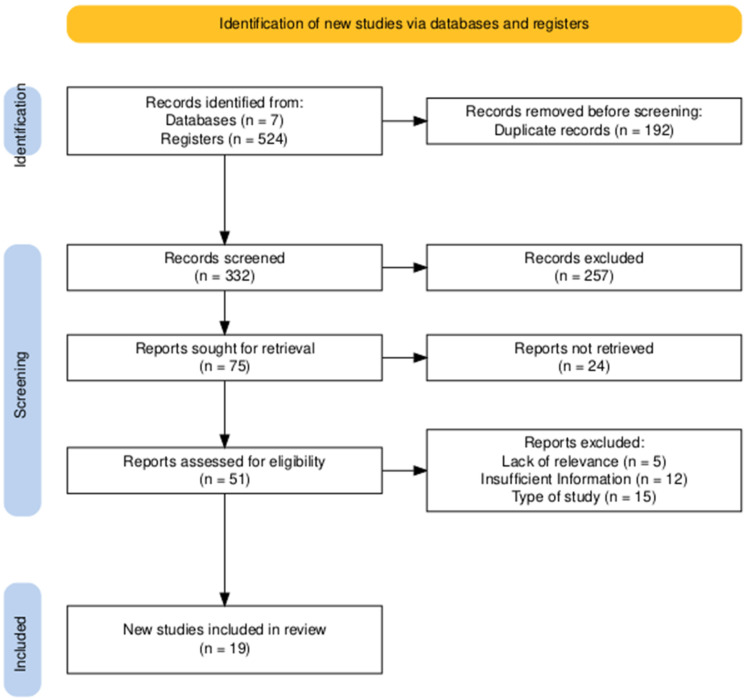
PRISMA diagram illustrating the study identification and screening workflow. An excellent level of agreement was achieved between reviewers. Cohen’s Kappa coefficients were 0.85 for the title and abstract review and 0.98 for the full-text eligibility assessment, reflecting a very high and consistent alignment throughout the selection process.

**Figure 2:**
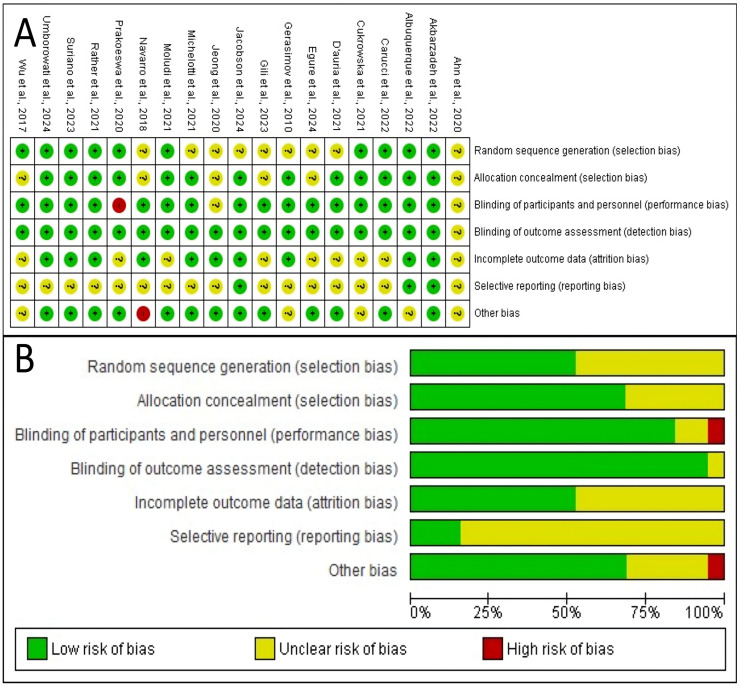
Risk of bias evaluation. (**A**) Judgments on the risk of bias for each included study in the seven evaluated categories. A green circle with a ‘+’ symbol stands for a low risk of bias, a yellow circle with ‘?’ indicates an unclear risk, and a red circle with ‘–’ denotes a high risk. (**B**) Summary plot illustrating the percentage of studies rated as having low, unclear, and high risk of bias for each analyzed category [22,23,24,25,26,27,28,29,30,31,32,33,34,35,36,37,38,39,40].

The PRISMA 2020 flow diagram (Figure 1) was generated using the online R version 4.3.0 package (https://estech.shinyapps.io/prisma_flowdiagram/), accessed on 15 January 2025). Figure 2 was created in R (version 4.3.0) using the ggplot2 package (https://cran.r-project.org/bin/windows/base/old/4.3.0/). Both resources were accessed between 15 January and 30 March 2025.

### 2.6. Risk of Bias Assessment

Two reviewers, F.S.D. and D.M.G., independently assessed the risk of bias for each study using the Cochrane Risk of Bias tool version 2 (RoB 2) [19]. They recorded their findings in Review Manager (RevMan) version 5.4, which was accessed between January 15 and March 30, 2025. The assessment covered five key domains: (1) the randomization process, (2) deviations from intended interventions, (3) missing outcome data, (4) measurement of the outcome, and (5) selection of the reported results.

Based on these domains, each study was rated as having a low risk, some concerns, or a high risk of bias. Any disagreements between the reviewers were resolved through discussion until they reached a consensus.

### 2.7. Assessment of the Quality of Evidence

The quality of the evidence was evaluated using a dual approach. The risk of bias for each individual study was assessed using the Cochrane Risk of Bias tool version 2 (RoB 2) [19], as detailed in Section 2.6. In parallel, the methodological quality of each clinical trial was scored using the Jadad scale [21]. This scale assigns a score from 0 to 5 based on descriptions of randomization, blinding, and handling of dropouts; higher scores indicate a higher-quality trial. It is important to note that no studies were excluded based on this score; it was simply taken into account when reporting the results. Overall evidence certainty: The overall certainty of the evidence for each outcome was rated using the Grading of Recommendations Assessment, Development and Evaluation (GRADE) approach. This method considers factors like study limitations (risk of bias), inconsistency, and imprecision. Based on the GRADE assessment, the final certainty of evidence for each outcome was classified into one of four levels: high, moderate, low, or very low.

### 2.8. Data Analysis

Statistical analyses were performed using R software version 4.4.1 (R Foundation for Statistical Computing, Vienna, Austria), accessed in March 2025, with the packages ‘meta’ (version 7.0-0) and ‘metafor’ (version 4.6-0). Forest plots and funnel plots were created using ‘ggplot2’ (version 3.5.1).

Two primary conditions were analyzed with their respective outcomes:

- Atopic dermatitis (12 studies, n = 817 participants): the primary outcome was the change in disease severity measured by the SCORing Atopic Dermatitis (SCORAD) index.
- Psoriasis (5 studies, n = 287 participants): outcomes included change in disease severity measured by the Psoriasis Area and Severity Index (PASI) and change in quality of life measured by the Dermatology Life Quality Index (DLQI).

For continuous outcomes, standardized mean differences (SMD) with 95% confidence intervals (CIs) were calculated using Hedges’ g to correct for potential small-sample bias. The inverse variance method was employed for pooling effect sizes. A random-effect model was fitted using the restricted maximum-likelihood (REML) estimator to calculate the between-study variance (τ2). The Hartung–Knapp adjustment was applied to obtain more conservative confidence intervals.

Heterogeneity was assessed using the Q statistic and quantified with the I^2^ statistic, where values around 25%, 50%, and 75% were considered as low, moderate, and substantial heterogeneity [19], respectively. Forest plots were generated to visualize individual study effects and the pooled estimate.

Funnel plots were created to visually assess publication bias. Formal testing for asymmetry using Egger’s test was conducted for the atopic dermatitis meta-analysis, as it included more than 10 studies (k = 12). This formal test was not performed for the psoriasis meta-analyses (k = 5) because such tests have low statistical power and can produce unreliable results with fewer than 10 studies.

Given the heterogeneity in measurement instruments and baseline severities across trials, standardized mean differences were essential to allow for a meaningful comparison. All statistical tests were two-tailed, with a significance level set at *p* < 0.05.

## 3. Results

### 3.1. Studies Identified for the Review

The study selection process is summarized in the PRISMA flow diagram (Figure 1). The initial literature search yielded a total of 524 records, identified through searches of 7 databases. After the removal of 192 duplicate entries, 332 unique records advanced to the title and abstract screening phase.

During this initial screening, 257 records were excluded by title and abstract. This left 75 reports that were deemed potentially relevant and were sought for full-text retrieval. Of these, 24 reports could not be retrieved, leading to a total of 51 full-text articles being thoroughly assessed for eligibility.

Upon detailed full-text review, a further 32 reports were excluded. The primary reasons for exclusion at this stage included inappropriate study design (n = 15), insufficient information for analysis (n = 12), and a lack of relevance to this review’s scope (n = 5). Ultimately, this rigorous selection process resulted in 19 studies being included in the final qualitative and quantitative synthesis.

The entire selection process was characterized by an excellent level of agreement between reviewers. The reliability of the screening and eligibility assessment was confirmed with Cohen’s Kappa coefficients of 0.85 for the title and abstract review and 0.98 for the full-text eligibility assessment, reflecting a very high and consistent alignment throughout.

### 3.2. Participant Characteristics

The characteristics of the participants in the 19 randomized clinical trials are summarized in Table 1. The studies encompassed a broad demographic and clinical spectrum, with sample sizes ranging from 30 participants in the trial by Prakoeswa et al. [22] to 154 in the study by Jacobson et al. [23]. Subject age varied significantly, covering populations from 4-month-old infants (Wu et al. [24]) to 70-year-old adults (Umborowati et al. [25]), reflecting the different dermatological conditions investigated, such as atopic dermatitis predominantly in pediatric populations and psoriasis or acne in adolescent and adult cohorts. Body mass index, when reported, was primarily in adult studies with psoriasis and generally indicated that participants were in normal-to-overweight ranges [26,27]. Baseline disease severity was assessed using a variety of validated, condition-specific instruments, such as SCORAD and EASI for atopic dermatitis, PASI for psoriasis, and AGSS for acne. Several studies also included a comprehensive set of secondary baseline parameters, such as quality of life questionnaires (IDQOL, DLQI), immunological markers (IgE, IL-4, IL-17, Foxp3), and biophysical skin properties (TEWL, hydration), providing a multifaceted characterization of the study populations before intervention.

**Table 1:** Participant characteristics.

Study Reference	Participants	Size (n)	Age (Years)	BMI (kg/m^2^)	Baseline Parameters
Gerasimov et al., 2010 [28]	Children with moderate-to-severe AD	90	1–3	Not Reported	SCORAD, IDQOL, DFI, topical steroid use, lymphocyte subsets
Rather et al., 2021 [29]	Children and adolescents with AD	90	3–18	Not Reported	SCORAD, IGA, IgE, ECP, eosinophils, CCL17, CCL27, skin moisture/sebum
Navarro et al., 2017 [30]	Children with moderate AD	50	4–17	Not Reported	SCORAD, topical steroid use
Wu et al., 2015 [24]	Children with AD (SCORAD ≥ 15)	66	0.3–4	Not Reported	SCORAD, steroid use, flare frequency, symptom-free duration
Jeong et al., 2020 [31]	Children with moderate AD	100 (66 analyzed)	1–12	Not Reported	SCORAD, ECP, IL-31, safety parameters
Michelotti et al., 2021 [32]	Adults with mild-to-severe AD	80	18–50	Not Reported	SCORAD, inflammatory cytokines (tape stripping), skin hydration, smoothness
Umborowati et al., 2024 [25]	Adults with mild-to-moderate psoriasis	49	18–70	24.8–27.4	PASI, DLQI, IL-10, IL-17, Foxp3
Cukrowska et al., 2021 [33]	Children with AD + CMPA	151	<2	Not Reported	SCORAD, specific and total IgE
Eguren et al., 2024 [34]	Patients with mild-to-moderate acne	81	12–30	Not Reported	AGSS, GAGS, lesion counts
Akbarzadeh et al., 2022 [35]	Adults with psoriasis (PASI > 2%)	52	18–60	Not Reported	PASI, DLQI, VAS
Albuquerque et al., 2022 [36]	Children/adolescents with AD	60	0.5–19	Not Reported	SCORAD, Hanifin & Rajka criteria, IgE, SPT
Carucci et al., 2022 [37]	Children with AD (ProPAD trial)	100	0.5–3	Not Reported	SCORAD, IDQOL, microbiome
Moludi et al., 2021 [38]	Adults with plaque psoriasis	50	18–50	Not Reported	PASI, PSS, DLQI, BDI-II, inflammatory and oxidative stress markers
D’Auria et al., 2021 [39]	Infants with moderate–severe AD	58	0.5–3	Not Reported	SCORAD, gut microbiota, cytokines, steroid use
Ahn et al., 2020 [40]	Children with mild-to-moderate AD	82	2–13	Not Reported	SCORAD, TEWL, IgE, cytokines, gut microbiome
Gilli et al., 2023 [26]	Adults with plaque psoriasis	35	>18 (Mean: 52.5)	Mean: 30.1	PASI, BSA, DLQI, IL-17, IL-23
Jacobson et al., 2024 [23]	Children and adults with AD	154	≥2	Not Reported	IGA, EASI, BSA, POEM, Pruritus NRS
Prakoeswa et al., 2020 [22]	Adults with AD	30	>14 (Mean: 37.9)	Not Reported	SCORAD, IgE, IL-4, IFN-γ, Foxp3+, IL-17
Suriano et al., 2023 [27]	Adults with plaque psoriasis	103	>18 (Mean: 51)	Mean: 29	PASI, DLQI

Abbreviations: AD: Atopic Dermatitis; CMPA: Cow’s Milk Protein Allergy; BMI: Body Mass Index; SCORAD: SCORing Atopic Dermatitis; IDQOL: Infant Dermatitis Quality of Life; DFI: Dermatitis Family Impact; IGA: Investigator’s Global Assessment; IgE: Immunoglobulin E; ECP: Eosinophil Cationic Protein; CCL17/27: Chemokines; PASI: Psoriasis Area and Severity Index; DLQI: Dermatology Life Quality Index; IL: Interleukin; Foxp3: Forkhead box protein P3; AGSS: Acne Global Severity Scale; GAGS: Global Acne Grading System; VAS: Visual Analog Scale; SPT: Skin Prick Test; PSS: Psoriasis Symptom Scale; BDI-II: Beck Depression Inventory-II; BSA: Body Surface Area; EASI: Eczema Area and Severity Index; POEM: Patient-Oriented Eczema Measure; NRS: Numeric Rating Scale; TEWL: Transepidermal Water Loss; IFN-γ: Interferon-gamma.

### 3.3. Intervention Characteristics

Details of the interventions and their respective comparison groups are presented in Table 2. Therapeutic approaches were markedly heterogeneous, including oral probiotics, synbiotics, postbiotics, and a topical live biotherapeutic. Formulations varied from single-strain products, such as *Lactobacillus rhamnosus* (Suriano et al. [27]), to complex multi-strain mixtures, as observed in the synbiotic formulation used by Akbarzadeh et al. [35], which combined eight bacterial strains with a prebiotic. Notably, some trials explored innovative strategies, such as the use of heat-inactivated postbiotics (*L. paracasei* CBA L74 in D’Auria et al. [39]) and a topical application of live *Roseomonas mucosa* (Jacobson et al. [23]). Doses for oral probiotics typically ranged between 1 × 10^9^ and 2 × 10^10^ CFU/day. Intervention duration was also variable, generally between 8 and 16 weeks, with one study extending to 6 months (Albuquerque et al. [36]). In all included trials, the active intervention was compared with a placebo that matched in pharmaceutical form, appearance, and taste, ensuring the integrity of the double-blind design.

### 3.4. Summary of Outcomes, Adherence, and Safety

Table 3 provides a summary of primary and secondary outcomes, along with adherence rates and reported side effects for each study. Primary outcomes showed a consistent pattern of clinical improvement in studies focused on atopic dermatitis, where a significant reduction in SCORAD scores was a frequent finding [22,28]. In contrast, the results for psoriasis were more varied, with some studies reporting significant decreases in PASI scores [35,38], while others found no statistically significant differences compared to placebo [27]. Secondary outcomes often included improvements in quality of life, measured with scales such as the DLQI, and changes in immunological markers; for example, Prakoeswa et al. [22] observed significant modulation of IL-4, IFN-γ, and Foxp3+ levels. Adherence to interventions was generally high in studies that reported it, with rates frequently exceeding 80% or 90%. The safety profile of interventions was consistently favorable; reported side effects were typically mild and transient, most commonly affecting the gastrointestinal system, with frequencies often similar to those observed in placebo groups.

### 3.5. Methodological Quality Assessment

Figure 2 provides a comprehensive overview of the methodological quality of the 19 studies included in this review. The analysis reveals that the overall risk of bias across these studies appears to be low to moderate, as no single domain is dominated by high-risk ratings and several domains show a high prevalence of low-risk ratings. However, a significant issue is the lack of clear reporting in certain key areas, indicated by the high percentage of “Unclear risk of bias” ratings. This suggests that while many studies may have been conducted properly, their authors often failed to provide enough detail to allow for a definitive verification of their methodological quality.

The methodological quality is strongest in several specific domains. The most robust area is the handling of incomplete outcome data, or attrition bias, where over 90% of studies were rated as low-risk. This increases confidence that the results are not skewed by participant dropouts. Furthermore, the blinding of outcome assessment (detection bias) and the blinding of participants and personnel (performance bias) are also areas of strength, with approximately 80% and 75% of studies rated at low risk, respectively. These strengths indicate that the results are less likely to be distorted by biases related to attrition or a lack of blinding.

Conversely, the analysis also highlights significant weaknesses, primarily stemming from areas where the risk of bias was unclear due to poor reporting. The most significant weakness is in allocation concealment (a form of selection bias), where the majority of studies were rated as having an unclear risk. This means the articles did not adequately specify how they concealed the allocation sequence from those enrolling participants. Selective reporting bias is another major area of concern, with roughly half the studies rated as unclear, making it difficult to determine if authors reported all pre-specified outcomes or only a selection.

The implications of this bias profile are significant for interpreting this review’s findings. The low risk in key areas like attrition and blinding lends credibility to the reported results. However, the primary limitation stems from the poor reporting that led to a high degree of unclear risk, especially for allocation concealment and selective reporting. This introduces a degree of uncertainty, as these biases cannot be confidently ruled out. Finally, as shown in Figure 2A, a few specific studies [22,30,40] have high-risk ratings, and their results should be interpreted with greater caution.

The methodological quality of the included trials, assessed using the Jaded scale, is presented in Table 4. The overall quality of the evidence is high, as all 19 studies achieved a score of 3 or higher (≥3), the threshold considered for high-quality research.

A significant majority, fourteen of the nineteen studies, obtained the maximum possible score of 5, indicating a robust methodological design and reporting. This high quality is largely attributable to the consistent implementation of key trial design elements. All included studies were described as randomized and double-blind, fulfilling the fundamental criteria for minimizing selection and performance bias. Furthermore, a high proportion of the trials provided an adequate description of the randomization and blinding methods, as well as a clear account of participant withdrawals and dropouts.

The remaining studies received scores of 3 or 4, indicating minor deficiencies, primarily in the detailed reporting of methodological procedures. For instance, the studies by Wu et al. [24] and Ahn et al. [40] did not provide sufficient detail on the method of randomization. Similarly, the trial by Akbarzadeh et al. [35] lacked a clear description of the blinding method. The studies by Gilli et al. [26] and Prakoeswa et al. [22] received the lowest score of 3, as they did not sufficiently detail the randomization method nor did they adequately describe participant withdrawals. These reporting gaps, while notable, were present in a minority of the trials.

### 3.6. Quantitative Analysis—Atopic Dermatitis

**Figure 3:**
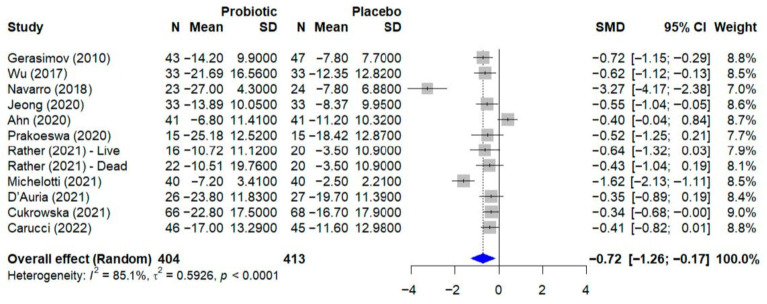
Forest plot of the effect of probiotics on the SCORAD score in atopic dermatitis. The meta-analysis of 12 studies [22,24,28,29,30,31,32,33,37,39,40] (N = 817) shows the pooled effect of probiotics compared to placebo. Using a random-effect model, probiotics achieved a statistically significant reduction in the SCORAD score (SMD = −0.72; 95% CI: −1.26 to −0.17; *p =* 0.015). Substantial heterogeneity was observed among the studies (I^2^ = 85.1%, 95% CI: 75.7–90.9%; Q-test: *p* < 0.0001). SMD, standardized mean difference; CI, confidence interval.

For the quantitative synthesis of atopic dermatitis outcomes, a meta-analysis was conducted including trials that evaluated disease severity using the SCORAD index as a continuous variable. From the initial set of studies on atopic dermatitis, a total of 12 trials were included in this analysis. The study by Jacobson et al. [23] was not included because it used the Eczema Area and Severity Index, or EASI, which is not directly comparable to the SCORAD score. Additionally, the trial by Albuquerque et al. [36] was excluded from the pooled analysis due to the absence of reported outcome data for the placebo group. The quantitative synthesis of outcomes for atopic dermatitis is presented in Figure 3. The meta-analysis, which included 12 studies with a total of 817 participants, evaluated the effect of probiotics on the SCORAD score. Using a random-effect model, the pooled results indicated that probiotic supplementation led to a statistically significant reduction in disease severity compared to placebo, with a standardized mean difference, or SMD, of −0.72 (95% CI, −1.26 to −0.17). Significant and substantial heterogeneity was observed among the studies (I^2^ = 85.1%, *p* < 0.0001), indicating considerable variability in the treatment effects across the included trials.

**Figure 4:**
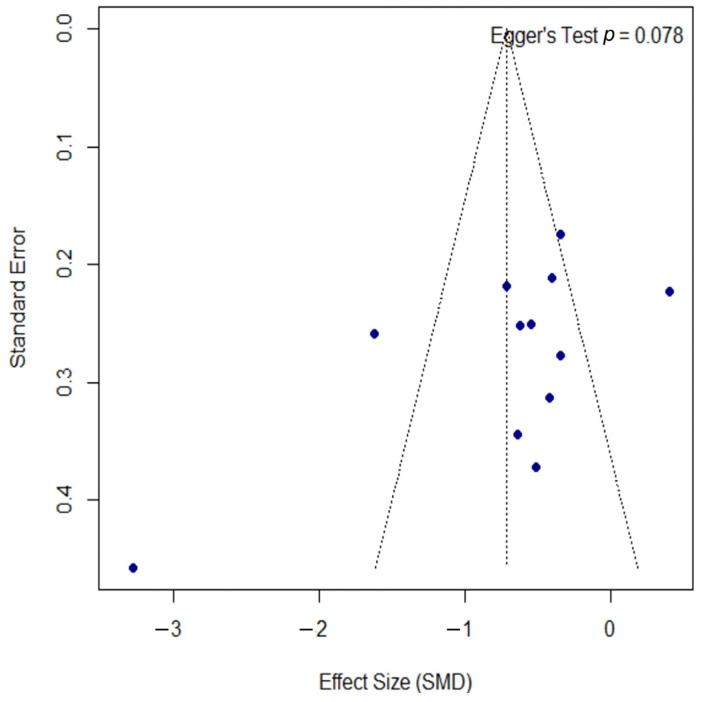
Funnel plot for the assessment of publication bias. The funnel plot was used to assess for publication bias. Each point represents an individual study, plotting its effect size (SMD) against the standard error. The formal Egger’s regression test did not indicate statistically significant asymmetry (t = −1.97, df = 10, *p =* 0.078), suggesting no strong evidence of publication bias. However, the *p*-value’s proximity to the 0.05 threshold may indicate a potential trend towards asymmetry. SMD, standardized mean difference; df, degrees of freedom.

Publication bias was assessed using a funnel plot, as shown in Figure 4. The plot displays some asymmetry; however, the formal Egger’s regression test did not indicate a statistically significant bias (*p* = 0.078). The proximity of this *p*-value to the significance threshold suggests a potential trend towards asymmetry, which should be considered when interpreting the overall findings.

### 3.7. Quantitative Analysis—Psoriasis

**Figure 5:**
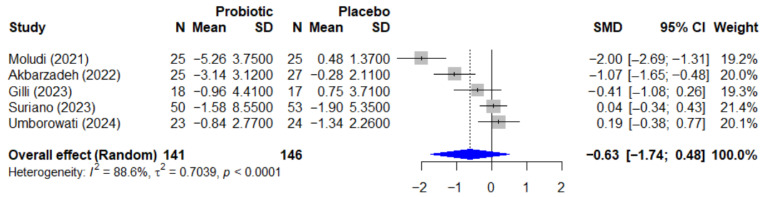
Forest plot of the effect of probiotics on the PASI score in patients with psoriasis. The meta-analysis of 5 studies [25,26,27,35,38] (N = 287) summarizes the effect of probiotics on the Psoriasis Area and Severity Index (PASI). The pooled effect, calculated using a random-effect model, was not statistically significant (SMD = −0.63; 95% CI: −1.74 to 0.48; *p =* 0.192). Very high heterogeneity was observed among the studies (I^2^ = 88.6%, *p* < 0.0001). SMD, standardized mean difference; CI, confidence interval; PASI, Psoriasis Area and Severity Index.

The quantitative analysis for psoriasis included 5 studies with a total of 287 participants. The meta-analysis of the effect of probiotics on the Psoriasis Area and Severity Index, or PASI, is presented in Figure 5. The pooled effect, calculated using a random-effect model, showed a standardized mean difference, or SMD, of −0.63 (95% CI, −1.74 to 0.48), which was not statistically significant (*p* = 0.192). This analysis was characterized by very high and significant heterogeneity among the studies (I^2^ = 88.6%, *p* < 0.0001).

**Figure 6:**
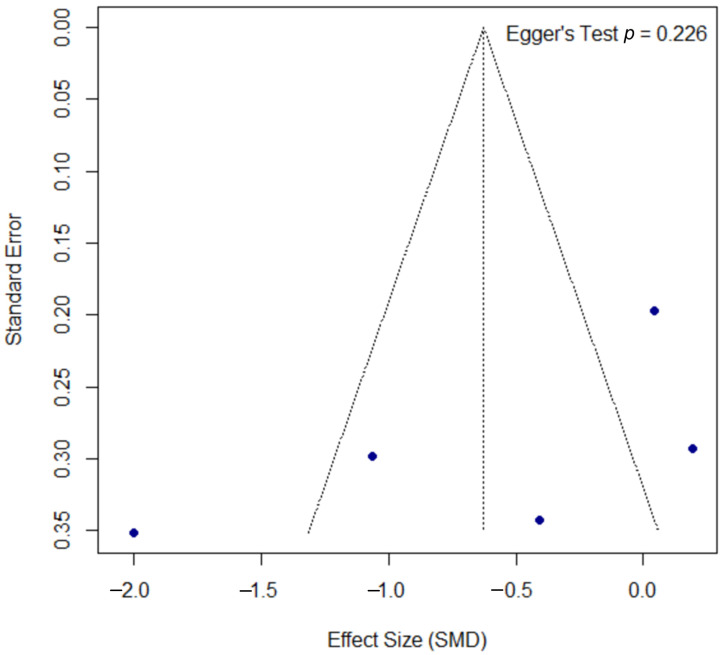
Funnel plot for the assessment of publication bias for the PASI outcome. The funnel plot of the effect size (SMD) against the standard error was used to assess for potential publication bias in the psoriasis meta-analysis. The formal Egger’s regression test did not find statistically significant asymmetry (t = −1.52, df = 3, *p =* 0.226), suggesting no strong evidence of publication bias. SMD, standardized mean difference; df, degrees of freedom.

The assessment for publication bias for the PASI outcome is shown in the funnel plot in Figure 6. A formal Egger’s test did not find statistically significant asymmetry (*p* = 0.226), suggesting no strong evidence of publication bias, though this interpretation should be approached with caution due to the low statistical power of the test with a small number of studies.

### 3.8. Quantitative Analysis—Analysis of Probiotics on Quality of Life

**Figure 7:**
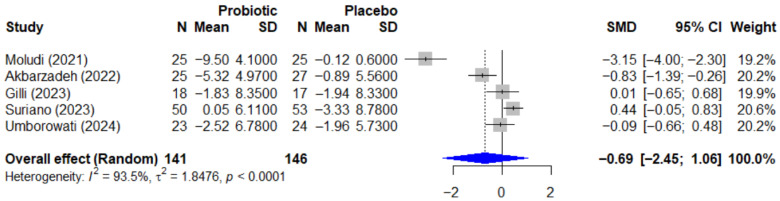
Forest plot of the effect of probiotics on quality of life (DLQI) in patients with psoriasis. This meta-analysis of 5 studies [25,26,27,35,38] (N = 287) summarizes the effect of probiotics on the Dermatology Life Quality Index (DLQI). The pooled effect, calculated with a random-effect model, was not statistically significant (SMD = −0.69; 95% CI: −2.45 to 1.06; *p =* 0.334). Extremely high and significant heterogeneity was observed among the studies (I^2^ = 93.5%, *p* < 0.0001), likely due to the conflicting directions of the individual study results. SMD, standardized mean difference; CI, confidence interval; DLQI, Dermatology Life Quality Index.

Regarding the quality-of-life outcome, the effect of probiotics on the Dermatology Life Quality Index, or DLQI, is summarized in the forest plot in Figure 7. The pooled effect was also not statistically significant, with an SMD of −0.69 (95% CI, −2.45 to 1.06, *p* = 0.334). This analysis revealed extremely high heterogeneity (I^2^ = 93.5%, *p* < 0.0001), which is likely attributable to the conflicting directions of the individual study results.

**Figure 8:**
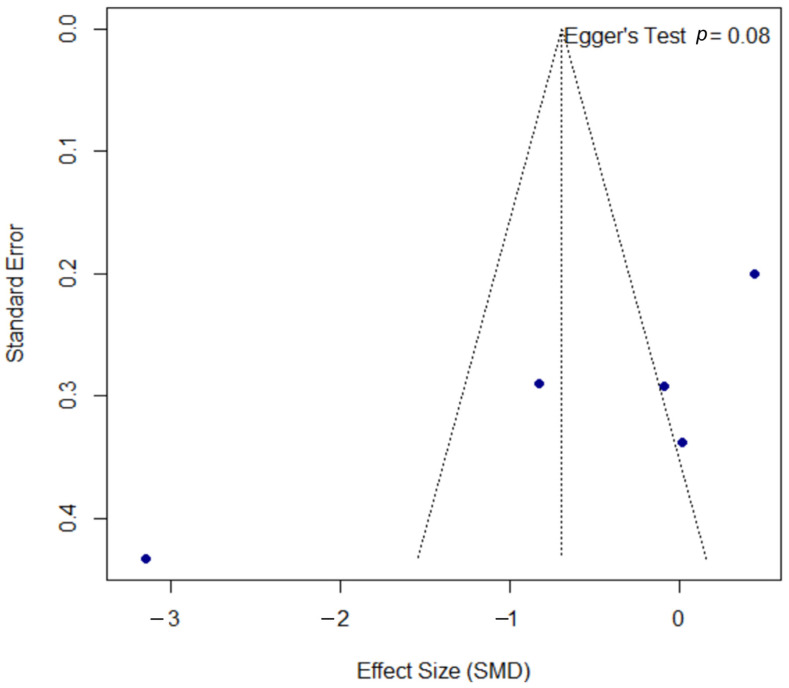
Funnel plot for the assessment of publication bias for the DLQI outcome. The funnel plot was used to assess for potential publication bias in the meta-analysis of the DLQI outcome. The formal Egger’s regression test did not reach statistical significance (t = −2.60, df = 3, *p =* 0.080). However, this borderline *p*-value may suggest a trend towards funnel plot asymmetry. This result should be interpreted with significant caution, as the power of the test is very low with only five included studies. SMD, standardized mean difference; df, degrees of freedom.

The funnel plot for the DLQI outcome, presented in Figure 8, was assessed for publication bias. Egger’s test did not reach statistical significance (*p* = 0.080); however, the borderline *p*-value may suggest a trend towards asymmetry, a finding that must be interpreted with significant caution given the limited power of the test with only five included studies.

## 4. Discussion

### 4.1. Main Findings

The results of this meta-analysis reveal a complex yet promising landscape for the use of microbial interventions in treating inflammatory dermatological diseases. For atopic dermatitis, a statistically significant reduction in SCORAD scores was found with probiotic use (SMD = −0.72; 95% CI: −1.26 to −0.17; *p* = 0.015), suggesting a moderate but consistent clinical benefit. This finding is particularly relevant considering that SCORAD is a validated and widely accepted tool for assessing atopic dermatitis severity, integrating both lesion extent and intensity [41].

However, the results for psoriasis present a more heterogeneous pattern. Although the meta-analysis did not reach statistical significance for the PASI index (SMD = −0.63; 95% CI: −1.74 to 0.48; *p* = 0.192), several individual studies demonstrated notable benefits. This variability could reflect fundamental differences in the pathophysiology of both conditions. While atopic dermatitis is characterized by a predominantly Th2 immune response, psoriasis involves more complex activation of Th1, Th17 pathways, and cytokines such as IL-17, IL-22, and IL-23, which may require more specific and targeted microbial strategies [42,43].

The high heterogeneity observed in the treatment effects across studies in both meta-analyses (I^2^ = 85.1% for atopic dermatitis and I^2^ = 88.6% for psoriasis) underscores the complexity of microbial interventions and the need for more standardized protocols. This variability can be attributed to multiple factors, including differences in strains used, administered doses, treatment duration, patients’ baseline characteristics, and evaluation methods employed. These findings collectively establish the foundation for understanding the mechanisms underlying these differential responses.

### 4.2. Mechanisms of Action of Probiotics, Prebiotics, and Synbiotics in Dermatology

Building upon these clinical observations, the mechanisms by which probiotics exert beneficial effects in dermatological diseases are multifaceted and operate through the gut–skin axis, a concept that has gained considerable attention in recent dermatological literature.

Systemic immunological modulation: Probiotics influence the immune system both locally and systemically. Recent research has demonstrated that Lactobacillus plantarum can significantly reduce the levels of proinflammatory cytokines such as IL-4, TNF-α, and IFN-γ while increasing the production of IL-10, a key anti-inflammatory cytokine [1].”In the context of atopic dermatitis, this is particularly relevant, as the condition is characterized by an exaggerated Th2 response with elevated levels of IL-4 and IL-13, which promote cutaneous inflammation and compromise barrier function [22,44].

Intestinal barrier strengthening: Probiotics strengthen intestinal barrier integrity through several mechanisms. They produce short-chain fatty acids (SCFAs) such as butyrate, acetate, and propionate, which are fundamental for maintaining intestinal epithelial integrity and reducing permeability [45,46,47]. A compromised intestinal barrier allows for translocation of bacterial antigens and toxins, triggering systemic inflammatory responses that can manifest in the skin.

Microbiome regulation: Probiotic species compete with pathogens for nutrients and adhesion sites, produce antimicrobial substances, and modulate intestinal microbiome composition. In patients with atopic dermatitis, dysbiosis characterized by reduced microbial diversity and increased potentially pathogenic species such as *Staphylococcus aureus* has been documented [48,49].

Prebiotic and synbiotic effects: Prebiotics, particularly fructo-oligosaccharides (FOSs) and galactooligosaccharides (GOSs), act as selective substrates for beneficial bacteria, promoting their growth and metabolic activity. Synbiotics, which combine probiotics and prebiotics, may offer synergistic effects. A recent study in psoriasis showed that synbiotic combination resulted in significant reductions in PASI scores and improvements in quality of life markers [35,50]. These mechanistic insights provide the theoretical framework for the clinical findings observed in our meta-analysis.

### 4.3. Comparison with the Existing Literature

The findings of this study show partial concordance with previous systematic reviews but also reveal important differences that merit detailed analysis, further contextualizing our results within the broader and most recent scientific evidence.

**Atopic dermatitis**: A comprehensive umbrella meta-analysis published in 2025 by Wang and Xu evaluated 38 meta-analyses encompassing 127,150 participants and found significant differences in SCORAD values favoring probiotics over placebo, with an effect size similar to that reported in our analysis. However, their subgroup analysis revealed that benefits were substantially more pronounced with synbiotic formulations, showing a weighted mean difference of −7.13 (95% CI: −9.83 to −4.43; *p* < 0.001) compared to probiotics alone, which demonstrated a more modest effect [51]. Notably, their analysis demonstrated that *Lactobacillus* species showed significant efficacy (WMD = −1.79; 95% CI: −3.47 to −0.11), while *Bifidobacterium* and prebiotics alone failed to demonstrate substantial benefits. A critical finding from this umbrella analysis was that therapeutic effects were limited to moderate-to-severe atopic dermatitis cases, with no significant improvement observed in mild presentations, suggesting that disease severity should be a key consideration in treatment selection.

**Psoriasis**: Contemporary meta-analyses focused on the gut–skin axis in psoriasis have reported markedly more optimistic results than those observed in our study. A systematic review and meta-analysis published in 2024 by Zhu et al. [52], which included 7 RCTs with 400 participants, demonstrated significant improvements in PASI scores with a mean difference of −3.09 (95% CI: −5.04 to −0.74; *p* = 0.01), contrasting with our non-significant findings. Although the percentage of patients achieving ≥75% reduction in PASI showed a favorable trend in the probiotic group (33.57% vs. 23.61%), this difference did not reach statistical significance (RR 1.40; 95% CI: 0.98–1.98; *p* = 0.06). Notably, their analysis revealed significant reductions in systemic inflammatory markers, particularly C-reactive protein levels (MD −2.36; 95% CI: −2.77 to −1.95; *p* < 0.0001), while changes in IL-6 levels were not statistically significant. The discrepancies between our findings and those of Zhu et al. for psoriasis may be attributed to differences in the included studies. Our search, concluded in March 2025, may have identified different trials or applied stricter inclusion criteria, leading to a more conservative estimate of the effect. Similarly, while our findings for atopic dermatitis align with the umbrella review by Wang and Xu, our focused analysis allows for a more granular examination of the specific evidence for this condition.

A comprehensive review by Thye et al. [53] examining the gut–skin axis in psoriasis provided additional mechanistic insights, reporting that *Lactobacillus* and *Bifidobacterium* species effectively reduced Th17-related cytokines and diminished psoriasis severity across multiple human studies. Their analysis highlighted cases of dramatic clinical improvement, including patients with severe pustular psoriasis achieving near-complete remission after *Lactobacillus sporogenes* supplementation and significant TNF-α reduction following *Bifidobacterium infantis* administration. These findings suggest that the therapeutic potential of microbial interventions in psoriasis may be strain-specific and depend on baseline inflammatory profiles, potentially explaining the heterogeneity observed across different studies including our own analysis.

**Quality of life**: A consistent finding across the contemporary literature is the improvement in quality-of-life indices, regardless of whether changes in clinical severity reach statistical significance. The Dermatology Life Quality Index, for example, showed significant improvements in multiple recent studies, with reported mean differences in scores ranging from −1.45 to −5.74 compared to placebo, suggesting that patient-perceived benefits may extend beyond objective changes in cutaneous lesions [26,38]. This phenomenon indicates that microbiome interventions may influence psychological and social dimensions of disease burden through mechanisms independent of visible skin improvement.

**Safety and tolerability**: Recent systematic reviews and meta-analyses have demonstrated the potential efficacy and safety of probiotics in treating psoriasis and atopic dermatitis. Probiotic supplementation has shown significant improvements in psoriasis severity, measured by the Psoriasis Area and Severity Index (PASI) [30,52]. For atopic dermatitis, probiotics, particularly Lactobacillus strains, have demonstrated effectiveness in reducing disease severity, especially in moderate-to-severe cases [54]. The safety profile of probiotics is favorable, with no significant difference in adverse events between probiotic and placebo groups [52,55].

**Strain and formulation heterogeneity**: Recent research highlights the potential benefits of multi-strain probiotic formulations over single-strain preparations. A network meta-analysis of preterm infant studies found that combinations of Lactobacillus and Bifidobacterium species were superior in reducing mortality and morbidity compared to single-strain probiotics [56]. Similarly, in adult patients with mild–moderate ulcerative colitis, combinations of specific Lactobacillus and Bifidobacterium strains showed greater efficacy in inducing clinical remission and reducing disease activity scores [57]. For pediatric food allergies, Lactobacillus GG demonstrated the most significant improvements in clinical outcomes and quality of life [58]. However, a review by Ouwehand et al. [59] found no conclusive evidence supporting the superiority of multi-strain over single-strain probiotics, emphasizing the need for structured research comparing their efficacy. These findings underscore the importance of further investigation into strain-specific effects and potential synergistic interactions in probiotic formulations.

**Predictive biomarkers**: The pursuit of personalized medicine in dermatological conditions has led to significant advances in identifying predictive biomarkers for probiotic interventions. Contemporary research demonstrates that treatment response heterogeneity can be partially explained through multi-dimensional biomarker approaches encompassing genetic, microbial, and metabolic factors [60]. In inflammatory bowel disease, which shares pathophysiological mechanisms with inflammatory skin conditions through the gut–skin axis, specific microbial signatures have emerged as robust predictors of therapeutic outcomes. Notably, the abundance of SCFA-producing taxa, particularly *Faecalibacterium* and *Roseburia* species, correlates positively with probiotic efficacy, while elevated levels of opportunistic pathogens such as *Enterobacteriaceae* predict poor response rates (Meade et al., 2023) [61].

Beyond taxonomic composition, functional metabolic profiling reveals that butyrate biosynthetic capacity and alternative metabolic pathways, including propionate and acetate production, serve as reliable indicators of treatment success (Meade et al., 2023) [61]. In the context of atopic dermatitis, host genetic factors play a crucial modulatory role in probiotic responsiveness. Filaggrin gene mutations (FLG), present in approximately 10% of European populations, significantly influence barrier function restoration and may require modified therapeutic approaches, including extended treatment duration or adjunctive barrier repair strategies [62]. These findings collectively support the development of precision medicine algorithms that integrate baseline microbiome profiling, genetic screening, and metabolic assessment to optimize probiotic selection and dosing regimens for inflammatory dermatological conditions.

### 4.4. Limitations of the Included Studies

Despite these promising findings, the results of this systematic review must be interpreted with caution due to methodological and practical limitations identified in the analyzed studies. First, the relatively small sample size in most trials, an average of 63.5 participants per study (range: 30–154), compromises statistical power to detect clinically relevant differences in outcomes such as atopic dermatitis exacerbations or hospitalizations. This limitation also prevents exploration of benefits in specific subgroups, such as patients with particular filaggrin mutations or severe forms of psoriasis.

The duration of the interventions varied widely, from 8 weeks (Prakoeswa et al. [22]) to 6 months (Albuquerque et al. [36]), with many being too short. This precludes a clear assessment of long-term effects, which is a major drawback for chronic skin conditions where immunological or microbiome shifts can take months or years to become apparent. This wide variability in study design, or heterogeneity, introduces inconsistencies that complicate meta-analyses and direct comparison of results.

Significant methodological shortcomings were a key concern. A notable 42% of studies provided inadequate details on their randomization process or allocation concealment methods (e.g., Gilli et al. [26]; Prakoeswa et al. [22]), creating a high risk of selection bias. Other studies introduced further bias through incomplete reporting of non-serious adverse events (e.g., Jacobson et al. [23]; D’Auria et al. [39]) or by failing to properly account for patient dropouts, which increases the risk of attrition bias (e.g., Cukrowska et al. [33]).

The interventions themselves were also highly variable, making it difficult to draw firm conclusions. Some studies used formulations with a single microbial strain (like *L. rhamnosus* or *B. lactis*), while others used complex, multi-strain combinations of up to eight species. This diversity, combined with a wide range of daily doses (from 1 × 10^9^ to 2 × 10^10^ CFU/day) and different delivery formats (capsules, powders, liquids), makes it challenging to attribute effects to specific strains or dosages.

Additional factors further limit the findings. For instance, no studies adjusted for confounding variables like the concurrent use of antibiotics or corticosteroids, nor did they perform stratification of patients by genotype or baseline disease severity. Furthermore, the generalizability of the evidence is restricted, as 58% of studies were conducted in specific populations (mainly Asian and European), and only six studies included adults, despite dermatological diseases affecting all age groups.

### 4.5. Limitations of the Review

Our review has several important limitations that should be considered when interpreting the findings. First, by including only studies published in English and Spanish, we introduced a potential language bias, possibly overlooking relevant research conducted in other languages. The risk of publication bias is heightened by the potential exclusion of unpublished studies, non-peer-reviewed trials (often called gray literature), or studies with negative results that remain absent from major databases. In addition to these selection-related issues, the studies we did include were very different from one another, as confirmed by high I^2^ values. This significant heterogeneity made it difficult to draw consistent, generalizable conclusions, and our subgroup analyses were insufficient to explain the observed variability. This high degree of variability, in turn, impacted our ability to perform a full meta-analysis for several clinically important endpoints, such as patient-reported quality of life, specific skin inflammation markers, and microbiome shifts due to the small number of methodologically similar studies. Furthermore, a robust assessment of publication bias was limited. For the psoriasis meta-analyses (k = 5), formal tests like Egger’s test have low statistical power, rendering their results unreliable. Even in the larger atopic dermatitis analysis (k = 12), Egger’s test yielded a borderline *p*-value (*p* = 0.078), as did the test for the DLQI outcome in psoriasis (*p* = 0.080). This pattern suggests that the risk of publication bias cannot be definitively ruled out, and the overall findings should be interpreted with appropriate caution. Taken together, these limitations underscore the need for future, well-designed research to build a stronger evidence base and refine clinical guidelines.

### 4.6. Clinical Implications

Based on the evidence and its limitations, several key implications for clinical practice emerge. Probiotics and synbiotics can serve as a valuable adjunct therapy for inflammatory skin diseases, acting as a complementary tool to improve care. For this approach to be successful, it must not replace fundamental treatments such as anti-inflammatory therapy or skin barrier repair. Adopting this strategy will require developing clear clinical guidelines that specify which patients are suitable candidates, along with standardized dosing and long-term follow-up protocols to properly evaluate their effects.

A key principle within these guidelines must be treatment personalization, as a one-size-fits-all approach is ineffective. Patients respond differently to specific strains due to a variety of factors, including their unique microbiome, age, disease progression, comorbidities, and genetic background (such as filaggrin mutations in atopic dermatitis). Optimizing treatment for an individual’s specific needs requires a collaborative, multidisciplinary team where dermatologists, gastroenterologists, immunologists, and nutritionists work together [63]. This team-based strategy also allows for closer clinical monitoring and treatment adjustments if a patient is not responding as expected; moreover, collaboration between specialties is key to conducting the large-scale trials needed to develop more specific guidelines on strain selection and treatment protocols. In practice, clinicians could consider these therapies for patients with mild-to-moderate atopic dermatitis, those with a history of gastrointestinal issues, individuals who respond suboptimally to conventional care, or those with an intolerance to standard systemic treatments [63,64].

From a safety perspective, the included trials suggest a strong safety profile, with no serious adverse events directly related to the interventions. However, the primary challenge to their effectiveness is patient adherence, as inconsistent use or discontinuation of the therapy can undermine its potential benefits [65,66]. Therefore, thorough patient education and ongoing support are essential to maximize treatment success and promote better overall control of inflammatory skin conditions.

### 4.7. Recommendations for Future Research

Future research in this field should include clinical trials with larger sample sizes and longer follow-up periods; we suggest six to twelve months or more. This would allow for a more definitive evaluation of changes in cutaneous barrier function, flare frequency, and quality of life parameters, as well as a clearer determination of the clinical effectiveness of probiotics or synbiotics in managing inflammatory dermatological diseases. Expanding study populations would also facilitate the application of the findings to a broader range of ages and disease stages.

Additionally, it is advisable to integrate state-of-the-art microbiome analysis techniques, such as metagenomics and 16S rRNA sequencing. These approaches would help clarify specific changes in intestinal and cutaneous bacterial composition and their correlation with key clinical parameters. Likewise, uniform measurement of systemic and cutaneous inflammatory markers, including IL-4, IL-17, IL-22, TNF-α, and barrier function biomarkers such as TEWL, would provide a more precise understanding of the relationship between microbiome modulation and inflammation.

Another vital consideration is the need for studies designed to compare specific strains, doses, and formulations, as well as evaluate synbiotics versus probiotics alone. Identifying the most effective formulation would determine whether combining probiotics with prebiotics or other nutritional supplements, such as SCFA precursors or antioxidants, has a synergistic effect. This work would contribute to optimizing therapeutic approaches in terms of both efficacy and safety.

It is essential to include additional clinically relevant endpoints to provide a more comprehensive view of the disease and interventions. These endpoints could encompass the following: (a) dermatology-specific quality of life measures (DLQI, POEM), (b) cutaneous barrier function evaluation (TEWL, hydration), (c) cutaneous microbiome analysis and its diversity, (d) specific inflammatory biomarkers (fecal calprotectin, serum cytokines), (e) topical treatment-free days, and (f) evaluation of allergic comorbidities.

Future studies should also focus on identifying predictive response biomarkers, which would enable precision medicine in probiotic use. Factors such as baseline microbiome composition, genetic polymorphisms in innate immunity-related genes, and cytokine profiles could serve as efficacy predictors.

Pharmacoeconomic studies are needed to evaluate the cost effectiveness of microbial interventions compared to standard therapies, particularly important given the increasing cost of biological treatments for moderate–severe psoriasis and atopic dermatitis. These evaluations would be crucial for implementation in public health systems and health policy decision-making. Implementation of these recommendations in future clinical trial design would provide more robust and clinically applicable evidence, facilitating the integration of microbiome-based therapies into standard dermatological practice.

## 5. Conclusions

This meta-analysis of 19 controlled trials (1104 participants) indicates that microbial interventions significantly improve atopic dermatitis (SMD = −0.72; *p* = 0.015), though the results for psoriasis are heterogeneous. These therapies, which act by modulating the gut–skin axis, are most effective when using multi-strain or synbiotic formulations. The high heterogeneity across studies (I^2^ > 85%) underscores the critical need to standardize strains, dosing, and treatment duration. Despite a generally favorable safety profile, advancing these therapies requires larger clinical trials and a personalized medicine approach to effectively integrate them as an adjunctive treatment in dermatology.

## Figures and Tables

**Table 2:** Intervention details and comparison groups.

Study Reference	Type of Probiotic, Prebiotic, or Synbiotic	Pharmaceutical Form	Dosage	Duration	Comparison Group
Gerasimov et al., 2010 [28]	*L. acidophilus DDS-1, B. lactis* UABLA-12 with FOS (Synbiotic)	Oral powder	10 × 10^9^ CFU/day	8 weeks	Placebo (rice maltodextrin)
Rather et al., 2021 [29]	*L. sakei proBio65* (Live and Dead cells)	Oral powder (sachet)	1 × 10^10^ cells/day	12 weeks	Placebo (microcrystalline cellulose) and dead cell group
Navarro et al., 2017 [30]	*B. lactis CECT 8145, B. longum CECT 7347, L. casei CECT 9104*	Oral capsule	1 × 10^9^ CFU/day	12 weeks	Placebo (maltodextrin)
Wu et al., 2015 [24]	*L. rhamnosus (MP108)*	Oral capsule	350 mg/day	8 weeks	Placebo (maltodextrin)
Jeong et al., 2020 [31]	*L. rhamnosus IDCC 3201 tyndallizate (Postbiotic)*	Oral capsule	1.0 × 10^10^ CPU/day	12 weeks	Placebo
Michelotti et al., 2021 [32]	*L. plantarum PBS067*, *L. reuteri PBS072*, *L. rhamnosus LRH020*	Oral capsule	3 × 10^9^ CFU/day (total)	56 days	Placebo (corn starch)
Umborowati et al., 2024 [25]	*L. plantarum IS-10506*	Oral capsule	2 × 10^10^ CFU/day	12 weeks	Placebo (both groups with topical standard treatment)
Cukrowska et al., 2021 [33]	*L. rhamnosus ŁOCK 0900, L. rhamnosus ŁOCK 0908, L. casei ŁOCK 0918*	Oral powder (sachet)	1 × 10^9^ CFU/day (total)	3 months	Placebo (maltodextrin)
Eguren et al., 2024 [34]	*L. rhamnosus CECT 30031* and *Arthrospira platensis*	Oral capsule	1 × 10^9^ CFU/day	12 weeks	Placebo (maltodextrin)
Akbarzadeh et al., 2022 [35]	*Lactocare^®^ (8-strain mix + FOS)* (Synbiotic)	Oral capsule	Not Specified	3 months	Placebo
Albuquerque et al., 2022 [36]	*L. rhamnosus, L. acidophilus, L. paracasei, B. lactis*	Oral powder (sachet)	1 g/day (4 × 10^9^ CFU total)	6 months	Placebo (maltodextrin)
Carucci et al., 2022 [37]	*L. rhamnosus GG* (LGG)	Oral capsule	1 × 10^10^ CFU/day	12 weeks	Placebo
Moludi et al., 2021 [38]	*L. acidophilus, B. bifidum, B. lactis, B. longum*	Oral drink	1.8 × 10^9^ CFU/day (total)	8 weeks	Placebo
D’Auria et al., 2021 [39]	*L. paracasei CBA L74* (Heat-killed, Postbiotic)	Fermented rice flour powder	8 g/day	12 weeks	Placebo (rice powder)
Ahn et al., 2020 [40]	*L. pentosus*	Oral	1.0 × 10^10^ CFU/day	12 weeks	Placebo
Gilli et al., 2023 [26]	*L. rhamnosus Lr-G14*	Oral capsule	5 × 10^9^ CFU/day	60 days	Placebo
Jacobson et al., 2024 [23]	*FB-401* (3 strains of *Roseomonas mucosa*) (Live Biotherapeutic)	Topical spray	1x 10^7^ CFU/mL	16 weeks	Placebo (sucrose solution)
Prakoeswa et al., 2020 [22]	*L. plantarum IS-10506*	Oral capsule (microencapsulated)	2 × 10^10^ CFU/day	8 weeks	Placebo (skim milk-Avicel)
Suriano et al., 2023 [27]	*L. rhamnosus ATCC 7469*	Oral liquid (whey formula)	6 × 10^5^ bacteria/mL	6 months	Placebo (whey formula)

Abbreviations: CFUs: Colony-Forming Units; FOS: Fructo-oligosaccharide; L.: *Lactobacillus; B.: Bifidobacterium;* AD: Atopic Dermatitis; FB-401: Oral probiotic formulation; LGG: *Lactobacillus rhamnosus* GG.

**Table 3:** Results, adherence, and side effects.

Study Reference	Primary Results	Secondary Results	Comparative Effects (vs. Control)	Adherence	Side Effects	Author’s Conclusions	Study Limitations	
Gerasimov et al., 2010 [28]	SCORAD reduction of 33.7%	IDQOL and DFI scores improved; reduced topical steroid use; CD4/CD25 decreased, CD8 increased	Probiotic > placebo (*p =* 0.001)	>90% in both groups	Similar frequency of AEs in both groups	Probiotic mix was associated with significant clinical improvement and corresponding lymphocyte changes	Absence of pretrial run-in period	
Rather et al., 2021 [29]	SCORAD score decreased in live (*p =* 0.0015) and dead cell (*p =* 0.0017) groups	Skin sebum content increased; IGA score decreased; eosinophil count decreased in live cell group	Live and dead probiotic > placebo for SCORAD (*p* < 0.05)	Not specified	No serious reactions reported	Both live and dead cells of	*L. sakei* proBio65 clinically improved AD symptoms	Short duration (12 weeks), small number of subjects per arm, limited biomarkers studied
Navarro et al., 2017 [30]	SCORAD reduction was 19.2 points greater in probiotic group	Significant reduction in topical steroid use in probiotic group	Probiotic > placebo (*p* < 0.001)	Not specified	No relevant adverse events reported	The probiotic mixture was effective in reducing SCORAD index and the use of topical steroids	Short follow-up, single center, not applicable to other populations/diets	
Wu et al., 2015 [24]	SCORAD change from baseline was −21.69 in probiotic group vs. −12.35 in placebo	No significant difference in topical corticosteroid use or symptom-free duration	Probiotic > placebo (*p =* 0.014)	>80% for per-protocol analysis	Similar AE rates in both groups		*L. rhamnosus* was effective in decreasing symptoms of AD after 8 weeks	Lack of long-term follow-up; lack of laboratory data (e.g., IgE, IL-4)
Jeong et al., 2020 [31]	SCORAD change was greater in probiotic group (−13.89 vs. −8.37)	ECP and IL-31 levels tended to decrease (significant in subgroup)	Probiotic > placebo (*p =* 0.0283)	Adherence rate was ~91% in both groups	No significant differences in safety parameters	Oral administration of RHT3201 showed therapeutic effect on AD	Could not completely stop TCS use; could not measure IFN-γ	
Michelotti et al., 2021 [32]	Significant decrease in SCORAD index in probiotic group	Improved skin smoothness, moisturization, and decreased inflammatory markers (TNF-alpha, TARC, TSLP)	Probiotic > placebo (*p* < 0.001) for SCORAD	No drop-outs reported	Well tolerated, no adverse events reported	Administration of selected probiotic strains resulted in a fast and sustained improvement in AD-related symptoms	Not specified by authors	
Umborowati et al., 2024 [25]	PASI score significantly reduced at week 6 and 12	DLQI scores were lower; IL-17 decreased, IL-10 and Foxp3 increased; lower probability of flares at 6 months	Probiotic > placebo for PASI (*p =* 0.049 at 12 wks)	47/49 completed the trial	Mild changes in defecation frequency and mild nausea		*L. plantarum* IS-10506 might effectively improve clinical outcomes and immune biomarkers in psoriasis	Small sample size, single-center study
Cukrowska et al., 2021 [33]	SCORAD decreased in both groups, but proportion of children with >30% improvement was higher in probiotic group	No significant difference in total IgE levels	Probiotic > placebo for proportion improved (OR 2.56, *p =* 0.012)	134/151 completed the 3-month intervention	Well tolerated, sporadic changes in stool consistency	The probiotic mixture offers benefits for children with AD and CMP allergy, especially in sensitized patients	Microbiome analysis not performed; adherence not systematically verified	
Eguren et al., 2024 [34]	50% of probiotic group improved in AGSS vs. 29.4% in placebo group	Significant reduction in non-inflammatory lesions; GAGS improvement was higher in probiotic group	Probiotic > placebo for AGSS improvement (*p =* 0.03)	74/81 completed the study	Number of AEs was similar in both groups	The probiotic used was effective and well tolerated for acne vulgaris patients	Diet not analyzed as a variable; results not applicable to populations outside 12–30 years	
Akbarzadeh et al., 2022 [35]	Significant decrease in PASI, VAS, and DLQI scores at week 12	100% of treatment group showed some PASI reduction vs. 7.4% of control group	Probiotic > placebo for PASI, VAS, DLQI (*p* < 0.05) at 12 wks	52 patients completed the study	Mild GI symptoms in a minority of patients	Oral administration of Lactocare^®^ resulted in the improvement in PASI, DLQI, and VAS scores	Small sample size due to COVID-19 pandemic dropouts	
Albuquerque et al., 2022 [36]	SCORAD decreased significantly more in the probiotic group	Probiotic group required topical immunosuppressant less frequently; no significant changes in IgE, SPT, or cytokines	Probiotic > placebo (*p* < 0.05) for SCORAD change	40/60 completed the study	Nausea, abdominal pain, pruritic episodes (more frequent in placebo group)	The probiotic mixture promoted a significant clinical response in children and adolescents with AD	Treatment duration may not have been optimal; small number of completers	
Carucci et al., 2022 [37]	Rate of subjects achieving MCID for SCORAD was higher in LGG group	IDQOL improved; beneficial modulation of gut (increased butyrate) and skin microbiome	Probiotic > placebo for achieving MCID (*p* < 0.05)	>80% consumption for per-protocol analysis	No adverse events reported	The probiotic LGG could be useful as adjunctive therapy in pediatric AD	Not specified by authors	
Moludi et al., 2021 [38]	Significant reduction in BDI, DLQI, PASI, and PSS scores	Significant increase in TAC and decrease in hs-CRP, IL-6, and MDA levels	Probiotic > placebo for all clinical scores and biomarkers (*p* < 0.05)	>90% compliance	Mild GI symptoms (12% placebo, 8% probiotic)	Probiotics improve patients’ quality of life and inflammatory biomarkers in psoriatic patients	Small sample size, short intervention period	
D’Auria et al., 2021 [39]	No significant difference in SCORAD change between groups	Significant steroid-sparing effect in the experimental group; no significant differences in cytokines or gut microbiota	No significant difference for SCORAD (*p =* 0.223)	53/58 completed the trial	No serious adverse events reported	Heat-killed	*L. paracasei* showed a steroid sparing effect, but was not effective in reducing AD severity	Not specified by authors
Ahn et al., 2020 [40]	No significant difference in SCORAD change between groups	Subjective SCORAD scores were significantly lower in the probiotic group for IgE sensitized AD subgroup (*p =* 0.019)	No overall difference vs. placebo. Significant for subjective score in sensitized subgroup	82/95 completed the study	Not specified	Could not find additional effects of	*L. pentosus* in AD, but may be effective in allergen-sensitized AD	Could not exclude intake of other fermented foods; baseline SCORAD difference; small subgroups
Gilli et al., 2023 [26]	Significant improvement in DLQI change (*p =* 0.035); reduction in PASI and BSA	No reduction in IL-17 and IL-23 levels	Probiotic > placebo for DLQI change. Numerical improvement in PASI/BSA	Adherence confirmed at day 30 and 60	More GI side effects in placebo group (64.3% vs. 35.7%)	Positive correlation between probiotic use and improvement in clinical aspects and scores	Limited number of patients, short follow-up period	
Jacobson et al., 2024 [23]	No significant difference in proportion of subjects achieving EASI-50	No significant differences in any secondary outcomes (EASI-75/90, IGA, BSA, Pruritus)	No difference vs. placebo (*p =* 0.7657)	~96% in both groups	Similar number of AEs; 1 unrelated SAE in probiotic group	FB-401 failed to prove superior to placebo but showed an acceptable safety profile	Inability to correlate with microbiome changes; restricted to mild–moderate population	
Prakoeswa et al., 2020 [22]	SCORAD score was significantly lower in the probiotic group at week 8	IL-4 and IL-17 decreased; IFN-γ and Foxp3+ increased; no significant change in IgE	Probiotic > placebo for SCORAD and immune markers (*p* < 0.05)	All 30 patients completed the study	Not specified		*L. plantarum* IS-10506 is effective for alleviating AD symptoms in adults owing to its immunomodulatory effects	Not specified by authors
Suriano et al., 2023 [27]	No significant change in PASI for experimental group (*p =* 0.105). Placebo group improved significantly (*p =* 0.019)	No significant change in DLQI for experimental group. Placebo group improved significantly (*p =* 0.031)	No significant difference between groups (*p =* 0.620)	Not specified	1 AE in experimental group vs. 4 in control group	Findings do not support the hypothesis that	*L. rhamnosus* produces clinical improvement in psoriasis	Single-center, hospital-based study; potential for placebo effect

Abbreviations: SCORAD: SCORing Atopic Dermatitis; IgE: Immunoglobulin E; IL: Interleukin; IFN-γ: Interferon-gamma; Foxp3^+^: Regulatory T cells with Foxp3 marker; EASI: Eczema Area and Severity Index; IGA: Investigator’s Global Assessment; PASI: Psoriasis Area and Severity Index; DLQI: Dermatology Life Quality Index; VAS: Visual Analogue Scale; BDI-II: Beck Depression Inventory-II; PSS: Psoriasis Symptom Score; GAGS: Global Acne Grading System; AGSS: Acne Global Severity Scale; BSA: Body Surface Area; QoL: Quality of Life; NR: Not Reported; AD: Atopic Dermatitis; GI: Gastrointestinal; SAE: Serious Adverse Event; MCID: Minimum Clinically Important Difference; TAC: Total Antioxidant Capacity; MDA: Malondialdehyde; hs-CRP: High-Sensitivity C-reactive Protein.

**Table 4:** Methodological quality assessment table (Jadad scale).

Author (Year)	Randomized Study? (1 Point)	Appropriate Randomization Method? (1 Point)	Double-Blind Study? (1 Point)	Appropriate Blinding Method? (1 Point)	Description of Withdrawals/Dropouts? (1 Point)	Total Jadad Score	Quality
Gerasimov (2010) [28]	Yes	Yes	Yes	Yes	Yes	5	High
Jeong (2020) [31]	Yes	Yes	Yes	Yes	Yes	5	High
Michelotti (2021) [32]	Yes	Yes	Yes	Yes	Yes	5	High
Navarro (2017) [30]	Yes	Yes	Yes	Yes	Yes	5	High
Rather (2021) [29]	Yes	Yes	Yes	Yes	Yes	5	High
Umborowati (2024) [25]	Yes	Yes	Yes	Yes	Yes	5	High
Wu (2015) [24]	Yes	NR	Yes	Yes	Yes	4	High
Ahn (2020) [40]	Yes	NR	Yes	Yes	Yes	4	High
Akbarzadeh (2022) [35]	Yes	Yes	Yes	NR	Yes	4	High
Albuquerque (2022) [36]	Yes	Yes	Yes	Yes	Yes	5	High
Carucci (2022) [37]	Yes	Yes	Yes	Yes	Yes	5	High
Cukrowska (2021) [33]	Yes	Yes	Yes	Yes	Yes	5	High
D’Auria (2021) [39]	Yes	Yes	Yes	Yes	Yes	5	High
Eguren (2024) [34]	Yes	Yes	Yes	Yes	Yes	5	High
Gilli (2023) [26]	Yes	NR	Yes	Yes	NR	3	High
Jacobson (2024) [23]	Yes	Yes	Yes	Yes	Yes	5	High
Prakoeswa (2020) [22]	Yes	NR	Yes	Yes	NR	3	High
Suriano (2023) [27]	Yes	Yes	Yes	Yes	Yes	5	High
Moludi (2021) [38]	Yes	Yes	Yes	Yes	Yes	5	High

The score ranges are 0–2 (very poor quality), 3–4 (moderate); and 5 (high quality). Studies with a score ≤ 2 are considered to be of low quality. NR = not reported.

## Data Availability

No new data were created or analyzed in this study.

## Abbreviations

The following abbreviations are used in this manuscript:

AD	Atopic Dermatitis
AGSS	Acne Global Severity Scale
AhR	Aryl Hydrocarbon Receptor
BMI	Body Mass Index
BSA	Body Surface Area
CCL17/27	Chemokines
CFUs	Colony-Forming Units
CI	Confidence Interval
CMPA	Cow’s Milk Protein Allergy
CSU	Chronic Spontaneous Urticaria
DLQI	Dermatology Life Quality Index
EASI	Eczema Area and Severity Index
ECP	Eosinophil Cationic Protein
FFAR2/3	Free Fatty Acid Receptor 2/3
FLG	Filaggrin
FOS	Fructo-oligosaccharide
GAGS	Global Acne Grading System
GPR41/43/109A	G Protein-Coupled Receptor 41/43/109A
GRADE	Grading of Recommendations Assessment, Development and Evaluation
HDAC	Histone Deacetylase
I-FABP	Intestinal Fatty Acid-Binding Protein
IGA	Investigator’s Global Assessment
IgE	Immunoglobulin E
IL	Interleukin
LBP	Lipopolysaccharide-Binding Protein
LGG	Lactobacillus rhamnosus GG
MCID	Minimum Clinically Important Difference
NRS	Numeric Rating Scale
PASI	Psoriasis Area and Severity Index
PICO	Population, Intervention, Comparison, Outcomes
POEM	Patient-Oriented Eczema Measure
PPARγ	Peroxisome Proliferator-Activated Receptor γ
PRISMA	Preferred Reporting Items for Systematic Reviews and Meta-Analyses
PROSPERO	International Prospective Register of Systematic Reviews
RCT	Randomized Controlled Trial
Reg3A	Regenerating Islet-Derived Protein 3-Alpha
REML	Restricted Maximum-Likelihood
RoB 2	Risk of Bias tool version 2
SCFA	Short-Chain Fatty Acid
SCORAD	SCORing Atopic Dermatitis
SMD	Standardized Mean Difference
SPT	Skin Prick Test
TEWL	Transepidermal Water Loss
Th1/Th2/Th17	T Helper Cell Type 1/2/17
TNF-α	Tumor Necrosis Factor-alpha
Treg	Regulatory T Cell
VAS	Visual Analog Scale
WoSCC	Web of Science Core Collection
